# A Retrospective Audit of the Timescales Involved in the Diagnosis and Management of Soft Tissue Knee Injuries at a Single National Health Service Trust: A Quality Service Improvement and Redesign Project

**DOI:** 10.7759/cureus.94641

**Published:** 2025-10-15

**Authors:** Ashmitha Vindya, Siddesh Bhushan Gangadharaswamy Nagabhushan

**Affiliations:** 1 Trauma and Orthopaedics, Ashford and St Peter’s Hospital National Health Service (NHS) Foundation Trust, Surrey, GBR

**Keywords:** mri, nhs, physiotherapy, quality improvement, service redesign, soft tissue knee injury

## Abstract

Objective

Acute soft tissue injuries are one of the most common presentations to the emergency department. There are many soft tissue knee injuries that might need surgery; therefore, prompt diagnosis of this injury ensures optimal management decisions at a minimal cost to the UK National Health Service (NHS). Despite this, regional and national variations in diagnosis and management exist, with anecdotal evidence of inefficiencies in the local patient pathway, which is not well established. This results in delays in diagnosis and treatment and inefficient use of imaging and knee clinics, affecting patient outcomes and causing unnecessary financial burden on the trust. To explore these factors further, a retrospective departmental audit of timescales from presentation to MRI diagnosis and definitive treatment decision was undertaken.

Methods

A retrospective audit at a single NHS trust was conducted, analysing all the referrals to the knee clinic over five months, from January 2023 to May 2023. This was done by collecting electronic and written patient records, and information on timescales involved in the diagnosis and management of each was compiled. Descriptive statistics were used to map each step of the pathway and timescales involved.

Results

A total of 346 patients were included; 77.7% (269/346) underwent MRI. Median waits were seven days from presentation to knee clinic, 33 days from MRI referral to scan, and 22 days from scan to review. The median diagnostic delay was 60 days (mean 56.6) from presentation to confirmed diagnosis, identifying MRI access and review as principal bottlenecks. Over half of the cohort (58.0%) waited longer than four weeks for an MRI, and only 10.3% had results reviewed within seven days. Among MRI scans, 42.8% showed normal/degenerative findings and 40.1% ligament/meniscal injury, highlighting a high utilisation of MRI with a relatively low yield. Surgery was required in 11.0% (median 128 days from presentation to operation), while 89.0% were managed conservatively, with physiotherapy initiated after a median delay of 70.5 days. Annualised observed costs were approximately £287,921 (consultations £182,688; MRI £105,233); scaled to an assumed 1,300 annual referrals, the pathway would cost ~£451,000 per year.

Conclusions

The current pathway demonstrates prolonged diagnostic delays (median 60 days), high MRI utilisation (77.7%) with low-yield imaging, and late rehabilitation initiation, all contributing to suboptimal patient outcomes and substantial cost. Introducing a physiotherapy-first triage model with criteria-based MRI referral and streamlined scan-to-review turnaround could significantly reduce diagnostic delays, improve functional recovery, and lower service expenditure across the NHS.

## Introduction

Soft-tissue knee injuries constitute a substantial proportion of urgent presentations in the United Kingdom, with estimates suggesting they account for a meaningful share of emergency attendances and that incidence has risen over the past two decades [1,2]. The burden is unevenly distributed, with injury patterns varying by age and sex, and participation in pivoting or high-demand sports conferring additional risk. Female athletes demonstrate a markedly higher rate of anterior cruciate ligament (ACL) injury than their male counterparts in comparable sport-related activities [3]. Moreover, sports participation and younger age were prominent features among affected populations [1]. These epidemiological characteristics, coupled with patient expectations for rapid return to activity, make timely and accurate diagnosis a core objective of high-quality musculoskeletal care. Timely diagnosis and optimal management of musculoskeletal injuries have become increasingly important in recent years in view of the ageing population and the rising prevalence of lifestyle-related health problems associated with inactivity [4,5].

Within our organisation, first-time soft-tissue knee presentations generate approximately 1,047 new knee-clinic referrals annually (2022-2023), underscoring the substantial diagnostic and therapeutic demand placed on musculoskeletal and imaging services. Contemporary pathways for assessment and management remain heterogeneous across the National Health Service (NHS). Reports from multiple centres describe variation in the timeliness of specialist review, access to magnetic resonance imaging (MRI) scans, and the interval to definitive decision-making, with consequent inequities in patient experience and use of resources [6]. In the acute setting, diagnostic uncertainty can be compounded by variable familiarity with specialist examination manoeuvres for ligamentous injuries among non-specialists, which may shift practice toward radiological confirmation and additional referrals, lengthening time to diagnosis and treatment planning [7]. Tools exist to steward imaging and direct the right patients to the right tests. The Ottawa Knee Rule (OKR) supports rational use of radiography after knee trauma and helps to limit unnecessary downstream investigations [8]. Even so, observational data suggest that only a minority of trauma clinic referrals ultimately represent serious ligamentous or meniscal pathology, implying that some clinic attendances and scans may be of low yield [9].

These observations have motivated interest in redesigning the front end of the pathway. Physiotherapy-led triage models, often drawing on advanced musculoskeletal physiotherapy practice and, in some settings, virtual fracture clinic (VFC) principles, have reported reductions in unnecessary MRI, shorter times to diagnosis, and more efficient streaming to orthopaedics when escalation criteria are met [10,11]. Within a selective-imaging strategy, MRI is reserved for patients with high surgical suspicion, mechanical symptoms, or failure to improve after an initial period of structured conservative care, aligning diagnostic resources with the probability of management change. Point-of-care ultrasound (POCUS) has emerging evidence in the accident and emergency department as an adjunct in the acute assessment of meniscal injury and may, in appropriately governed services, complement clinical examination to inform early decision-making [12].

To standardise care, national frameworks set time-based and referral criteria benchmarks. Under the NHS Constitution, the Referral-to-Treatment (RTT) standard requires 92% of consultant-led pathways to be completed within 18 weeks, while the Diagnostics (DM01) standard mandates that < 1% of patients should wait longer than six weeks for MRI [13,14]. Yet, national data show persistent breaches: as of January 2025, only 58.9% of RTT pathways met the 18-week target, and approximately 6% of patients waited > 6 weeks for an MRI [14]. At the same time, large UK-based cohort analyses have shown that only around 22% of MRI-confirmed isolated meniscal tears proceed to arthroscopic surgery, emphasising the importance of selective imaging and careful triage [15].

Against this backdrop, clinicians in our trust perceived avoidable delays at several steps, particularly around MRI access, the turnaround from scan to review, and the late initiation of rehabilitation in non-operative cases. These had potential implications for clinical outcomes, patient satisfaction, and service cost. We therefore undertook a retrospective audit to map the patient journey for first-time soft-tissue knee injury referrals from initial presentation through diagnosis to definitive management within our organisation. Our objectives were to (i) quantify interval timings at each stage of the pathway, (ii) characterise imaging utilisation and diagnostic yield, (iii) describe management outcomes, and (iv) estimate associated resource use.

## Materials and methods

Study design and setting

This study was conducted as a retrospective audit of consecutive new referrals to the acute knee clinic at a single National Health Service (NHS) trust. The audit period covered all clinic attendances between January and May 2023.

Eligibility criteria

Patients were considered eligible if they were attending the clinic for the first time with a documented soft-tissue knee injury. Exclusion criteria included patients presenting with fractures, those who had undergone previous knee surgery, follow-up attendances for chronic knee disease, and cases with incomplete records in which essential chronological data required for analysis were unavailable.

Data sources and collection

Patient data were extracted from the electronic health record systems Cerner and Evolve. Information collected included the referral source, date of initial presentation, and all subsequent chronological milestones along the care pathway. These milestones comprised the date of knee clinic attendance, the date of MRI referral, the date on which the MRI scan was performed, the date of MRI review, and the date of definitive management, defined as either surgical intervention or initiation of physiotherapy or other conservative management. MRI findings were categorised as normal or degenerative, ligamentous or meniscal, bony or contusion-related, or other findings. Management strategies were classified as surgical or conservative, and the timing of physiotherapy initiation was also recorded. Unit costs were obtained from internal NHS finance datasets and were set at approximately £220 for each new consultation and £163 for each MRI scan.

Outcome measures

From these data, several time intervals were derived. These included the time from presentation to knee clinic attendance, the interval between MRI referral and MRI acquisition, the interval between MRI acquisition and MRI review, and the overall time from initial presentation to diagnosis. Diagnosis was defined as the date of MRI review, where available, or the date of knee clinic attendance in cases where an MRI was not performed. In addition, the total time from initial presentation to definitive management, defined as either surgical intervention or commencement of physiotherapy/conservative care, was calculated.

Statistical analysis

Data were analysed using descriptive and non-parametric statistical methods. Continuous variables, such as pathway intervals, were summarised using medians, interquartile ranges (IQRs), and means to account for non-normal right-skewed distributions typical of healthcare waiting-time data. Categorical variables, including referral sources, imaging utilisation, and management outcomes, were reported as frequencies and percentages. Kruskal-Wallis tests were performed to compare median waiting times across referral sources for key pathway phases. Significant intergroup differences were defined as p < 0.05. The H-statistic was reported to indicate the magnitude of between-group rank variance. For example, marked variation was seen in early pathway access between presentation and clinic attendance (H = 66.66, p < 0.001) and presentation to diagnosis (H = 45.62, p < 0.001) but not for later MRI-related intervals (H = 6.80, p = 0.56; H = 8.65, p = 0.37).

These findings demonstrate that the referral source was a key determinant of early access to specialist review and diagnostic confirmation, whereas downstream imaging and reporting processes were more uniform once MRI was requested. Descriptive cost modelling was undertaken to estimate service expenditure. Unit costs were applied to new consultations (£220) and MRI scans (£163). The total observed cost for the five-month cohort was calculated and annualised using a multiplication factor of 2.4 to estimate a 12-month equivalent. A secondary scenario extrapolated costs to a projected annual caseload of 1,300 referrals (reflecting recent clinic activity of 1,047-1,300 new cases per year) to inform service-planning comparisons under existing utilisation rates. All analyses were performed using standard spreadsheet-based computation and cross-checked manually to ensure internal consistency. Formal multivariate modelling was not pursued, as the audit objective was exploratory pathway mapping rather than hypothesis testing.

## Results

Demographics

A total of 346 patients were included in the analysis. The median age was 43 years (interquartile range [IQR]: 34 years), with an age range of 6 to 86 years. The cohort demonstrated a nearly equal gender distribution, comprising 179 males (51.7%) and 167 females (48.3%), yielding a male-to-female ratio of approximately 1.07:1. This demographic spread reflects a broad spectrum of presentations, encompassing both younger, sport-related soft-tissue knee injuries and older, degenerative cases typically encountered in NHS musculoskeletal clinics. This is demonstrated in Table 1.

**Table 1:** Demographic characteristics of the study population (n=346) The table summarises baseline demographic data of all patients included in the audit cohort. The median age was 43 years (IQR = 34 years), ranging from 6 to 86 years. The gender distribution was nearly equal, with 179 males (51.7%) and 167 females (48.3%), yielding a male-to-female ratio of 1.07:1. This distribution reflects the diverse case mix encountered in NHS musculoskeletal clinics, spanning both sport-related soft-tissue injuries in younger patients and degenerative presentations in older adults.

Variable	Result
Sample size (n)	346
Median age	43 years
Interquartile range (IQR)	34 years
Age range	6-86 years
Male	179(51.7%)
Female	167(48.3%)
M:F ratio	1.07:1

**Figure 1:**
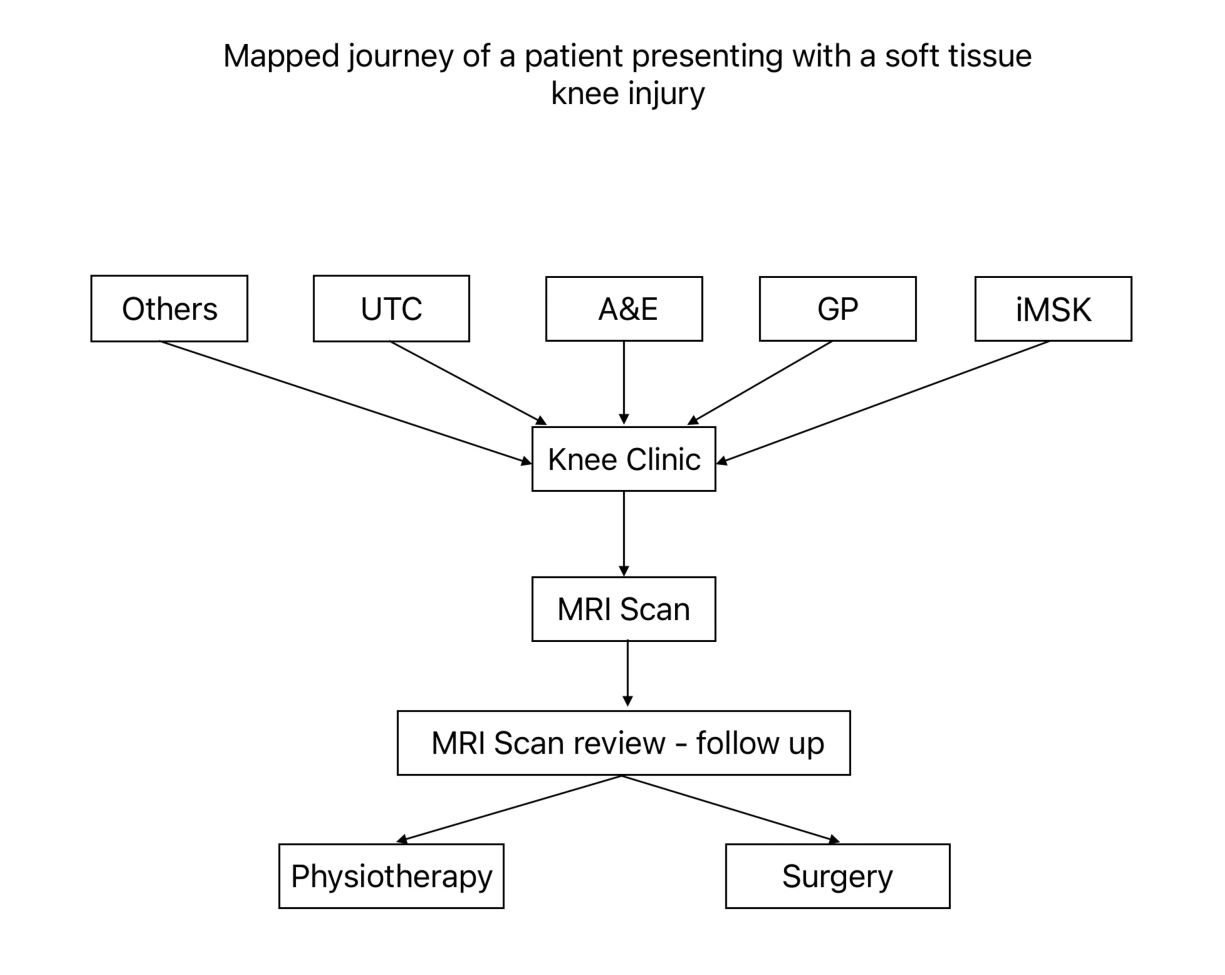
Patient pathway from initial presentation to definitive management in soft-tissue knee injury The diagram illustrates the referral routes to the knee clinic (urgent treatment centre [UTC], accident and emergency [A&E], general practitioner [GP], integrated musculoskeletal service [iMSK], and other sources). Subsequent stages include MRI scanning, MRI review at follow-up, and eventual definitive management through physiotherapy or surgery. Figure created by the authors for this study.

Across the audit window (January-May 2023), 346 new referrals to the acute knee clinic met the inclusion criteria. Referral origins were UTC 37.9% (131/346), ED 23.4% (81/346), GP 22.8% (79/346), knee clinic 6.6% (23/346), walk-in 5.8% (20/346), physiotherapy 2.3% (8/346), and other single-digit sources. The mapped journey of a patient presenting with a soft-tissue knee injury through the referral and management pathway is illustrated in Figure 1. This distribution confirms that urgent and emergency streams account for 61% of patient flow, consistent with the front-door emphasis in the pathway mapped. 

Diagnostic intervals along the pathway

Time to diagnosis was decomposed into three sequential phases: (i) presentation to the knee clinic (access to specialist triage), (ii) MRI referral to MRI scan (diagnostic acquisition), and (iii) MRI scan to MRI review (reporting and clinician review turnaround). The final diagnosis was defined as the date of MRI review, where applicable, or the date of first knee clinic attendance in patients not undergoing MRI. The median and mean durations for each phase, along with overall pathway intervals, are summarised in Table 2.

**Table 2:** Diagnostic and management intervals across the soft tissue knee injury pathway The table summarises time intervals at key stages of the patient pathway from initial presentation to definitive management. The median waiting time from presentation to knee clinic was seven days (IQR = 12). Following MRI referral, the median delay to MRI acquisition was 33 days (IQR = 30), with a further 22 days (IQR = 22) until MRI review. The overall median time from presentation to diagnosis was 60 days (IQR = 58), and from presentation to definitive management was 97 days (IQR = 90). These findings highlight substantial variation in diagnostic and treatment timelines, particularly around MRI access and reporting phases within the NHS musculoskeletal pathway.

Interval	N	Median (days)	IQR (Days)
Presentation to knee clinic	341	7	12
MRI referral to MRI Scan	269	33	30
MRI Scan to MRI review	255	22	22
Presentation to Diagnosis	343	60	58
Presentation to definitive management	159	97	90

Across the pathway, the median overall diagnostic interval from initial presentation to confirmed diagnosis was 60 days. This indicates that most patients experienced an 8-9-week delay before diagnostic confirmation, with the largest proportion of this delay attributable to MRI acquisition and review bottlenecks rather than front-end access to the knee clinic.

Presentation to Knee Clinic (Access to Specialist Triage)

The median interval was seven days, representing the shortest step in the pathway. However, the large discrepancy between mean and median suggests wide variability, indicating a long tail of patients with slower access to the weekly clinic, aligning with the observation that clinic slots. 

MRI Referral to MRI Scan (Diagnostic Acquisition Bottleneck)

Among patients who underwent MRI, the median time from referral to scan was 33 days (mean 33.7). A total of 58.0% waited longer than four weeks, with only 11.9% scanned within one week and 7.8% within two weeks. This interval was the principal contributor to diagnostic delay, highlighting restricted MRI capacity and scheduling constraints.

MRI Scan to MRI Review (Reporting and Clinician Review Turnaround)

After excluding implausible intervals, the median delay between scan and review was 22 days. Only 10.3% of cases were reviewed within seven days and 26.2% within 14 days, indicating a typical turnaround of two to three weeks. These post-scan review delays compounded upstream bottlenecks and further prolonged the time to definitive planning.

Overall Diagnostic Delay and Completion of Care Pathway

When combined, these intervals produced a median diagnostic delay of 60 days. The median time from presentation to definitive management, defined as surgical intervention or initiation of physiotherapy/conservative care, was 97 days. This equates to roughly three months from first presentation to active treatment initiation. The long right-tailed distribution reflects substantial variation, with many patients awaiting MRI, review, or surgical listing at the time of audit completion. 

Significant variation was observed in the early stages of the patient pathway, particularly between presentation and knee clinic attendance (H = 66.66, p < 0.001) and between presentation and diagnosis (H = 45.62, p < 0.001). These findings indicate that the referral source exerts a strong influence on initial access to specialist review and subsequent diagnostic confirmation. In contrast, the MRI-related intervals from referral to scan (H = 6.80, p = 0.56) and from scan to review (H = 8.65, p = 0.37) did not demonstrate significant intergroup differences, suggesting that once an MRI is requested, imaging acquisition and reporting processes are relatively uniform across sources. Overall, the results highlight front-end variability in access and triage, while downstream processes (imaging and review) appear more standardised within the trusts' operational framework. This has been demonstrated in Table 3.

**Table 3:** Statistical comparison of waiting times between referral sources (Kruskal–Wallis test) This table presents the results of the Kruskal–Wallis H test used to compare waiting times across different referral sources at key stages of the patient pathway. A highly significant difference was observed in the “Presentation to knee clinic” interval (H = 66.66, p = 2.26×10⁻¹¹) and the overall “Presentation to diagnosis” interval (H = 45.62, p = 2.81×10⁻⁷), indicating substantial variability between referral origins. No statistically significant differences were found for the “MRI referral to MRI scan” (p = 0.56) and “MRI scan to MRI review” (p = 0.37) intervals. These findings suggest that the referral source primarily influences early access to specialist assessment rather than downstream imaging stages.

Interval	Kruskal – Willis H	p-value	Interpretation
Presentation to knee clinic	66.66	2.26 x 10^-11^	Highly significant difference between referral sources
MRI referral to MRI Scan	6.8	0.56	No significant difference
MRI Scan to MRI review	8.65	0.37	No significant difference
Presentation to Diagnosis	45.62	2.81 x 10^-7^	Significant difference between referral sources

Imaging utilisation and diagnostic yield

**Figure 2:**
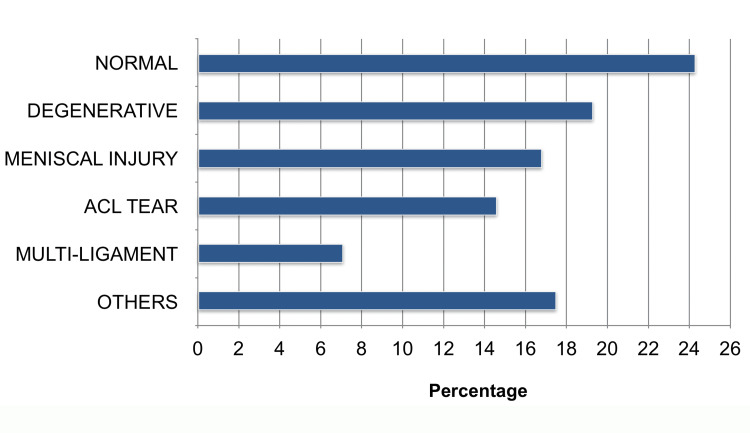
Distribution of MRI findings in patients with soft-tissue knee injuries The chart demonstrates the relative proportions of MRI outcomes within the study cohort. Categories include normal degenerative changes, meniscal injury, anterior cruciate ligament (ACL) tear, multi-ligament injury, and other findings. Figure created by the authors for this study.

Overall, MRI utilisation was 77.7% (269/346). Among completed MRIs, 42.8% reported normal/degenerative findings, 40.1% ligament/meniscal pathology, 14.1% other findings, and 1.5% bony/contusion. The distribution of MRI findings among patients with soft-tissue knee injuries is presented in Figure 2. 

The high proportion of low-yield scans supports your prior contention that MRI is sometimes used as a screening rather than a confirmatory tool in non-surgical presentations.

Management decisions and downstream timing

Surgery occurred in 11.0% (38/346). Presentation to having surgery had a median of 128 days (mean 200; IQR ≈87-325; range 18-470), underscoring the prolonged journey for the minority requiring operative care once diagnostic and listing processes are considered. Conservative management was used in 89.0%. Among non-operative patients with recorded therapy dates, the median time from clinic to physiotherapy had a median of 70.5 days (mean 65.9; n=122); 7.2% initiated physiotherapy within 30 days of the clinic appointment. The median remains indicative of systematic delay in rehabilitation onset, which mirrors the diagnostic lags upstream.

Differential performance by referral route

Median diagnosis times were shortest for UTC (53.5 days) and ED (58.0 days) and longest for GP (74.0 days). Concomitantly, MRI rates were lower in UTC (73.3%) and ED (79.0%) versus GP (83.5%). This suggests front-door triage pathways (UTC/ED) are associated with earlier diagnosis and slightly more selective imaging, whereas GP-routed patients accrue longer waits, likely reflecting added steps and re-triage prior to imaging and review.

Resource use and costs

Using internal unit costs (£220 per new consultation; £163 per MRI), the observed five-month cohort cost £119,967 (consultations £76,120; MRI £43,847). Annualised, this equates to ~£287,921 (consultations £182,688; MRI £105,233). For comparability with your service-planning denominator, scaling to 1,300 referrals/year at the observed 77.7% MRI rate yields ~£286,000 (consults) plus ~£165,000 (MRI) ≈ £451,000, aligning with your prior estimate of the annual burden under the current pathway.

## Discussion

This audit identifies prolonged diagnostic timelines, high MRI utilisation with substantial low-yield scans, and late access to rehabilitation across our local soft-tissue knee pathway. UTC/ED referrals reached diagnosis approximately 16-20 days earlier (medians 53-58 days) than GP (74 days) and had lower MRI rates. This suggests the front-end triage and referral pathway materially influences downstream efficiency, supporting the case for standardised streaming and physiotherapy-led rapid access across all entry points, including GP. The median 33-day interval from MRI referral to scan, followed by a 22-day scan to review the median, culminates in a mean 56.6-day journey from presentation to diagnosis, a cadence that risks deconditioning, persistent pain, and dissatisfaction. Despite guidance on selective imaging, MRI was performed in ~78% of cases, yet ~43% of reports were normal/degenerative, unlikely to change management in most scenarios, and consistent with concerns that MRI is sometimes used as a screening rather than a confirmatory tool. Concentrating MRI on patients with red flags, mechanical symptoms, high surgical suspicion, or failed six-week conservative care would likely cut imaging demand without compromising safety.

Delays of the magnitude observed (median ≈60 days to diagnosis and ≈10 weeks to rehabilitation start in nonoperative cases) are likely to exacerbate pain, deconditioning, fear-avoidant behaviours, and time away from work or sport, and may degrade patient-reported outcomes during the subacute period. For ACL-spectrum injuries in particular, prolonged uncertainty and delayed rehabilitation increase the risk of muscle atrophy, proprioceptive deficits, and persistent instability, which can prolong the overall recovery trajectory even when surgery is not ultimately required [6,7]. For meniscal pathology, delayed functional loading and unresolved mechanical symptoms may entrench compensatory movement patterns, complicating later rehabilitation. Our data show that the minority who progress to surgery (11.0%) wait a median of 128 days from presentation, reflecting both diagnostic and listing workflows. This timeline underscores the importance of early, structured rehabilitation to mitigate strength and function loss while patients await definitive management. By contrast, earlier physiotherapy emphasised in physiotherapy-first models has been associated with faster functional recovery, lower downstream imaging, and higher satisfaction in comparable NHS services [10,11,12,16]. Only 7.2% began physiotherapy within 30 days of the knee clinic review; the median was 70.5 days. Given that 89% were managed non-operatively, this constitutes a major opportunity: earlier physiotherapy should be the rule, not the exception. Hence, physiotherapy-first triage with rapid education and graded activity, escalating to AKSS/VFC/orthopaedics for defined criteria (e.g., mechanical locking, frank instability).

Alignment with national guidance

The findings of this audit highlight several divergences from national guidance on the assessment and management of soft-tissue knee injuries. NICE Clinical Knowledge Summaries (CKS) recommend that MRI should not be performed routinely in primary care and is most appropriate in secondary care for suspected ligamentous or meniscal injury after clinical assessment [17]. Similarly, the Evidence-Based Interventions (EBI) programme, endorsed by the Academy of Medical Royal Colleges, advises that MRI should not be requested for suspected degenerative meniscal tears unless mechanical symptoms are present or conservative treatment has failed [18]. Despite this, MRI utilisation in our cohort was 77.7%, with 42.8% showing only normal or degenerative findings, suggesting overuse of imaging outside the intended criteria.

National improvement frameworks also emphasise early conservative management and physiotherapy-led triage. The NHS England Musculoskeletal (MSK) and Orthopaedic Referral Optimisation Framework promotes a “right person, right place, right time” model, encouraging community-based MSK services and first-contact practitioner (FCP) triage before imaging or secondary-care referral [19]. In contrast, only 7.2% of patients in our cohort commenced physiotherapy within 30 days of clinic review (median 70.5 days), indicating delayed rehabilitation access. Furthermore, national pathways advocate expedited imaging for acute ligament injuries when clinically justified. BOA/BOAST and regional MSK guidance recommend MRI within two weeks where the diagnosis remains uncertain, with reporting and review ideally within 7-14 days [20]. In this study, the median intervals of 33 days from referral to MRI and 22 days from scan to review (overall median diagnostic delay = 60 days) substantially exceeded these standards, reflecting a diagnostic bottleneck that could delay definitive management.

Overall, these findings suggest that the current pathway diverges from NICE and NHS improvement recommendations by over-utilising MRI, under-utilising physiotherapy-first triage, and exceeding expected diagnostic timelines. Realignment with national frameworks through criteria-based MRI referral, early physiotherapy access, and targeted fast-track imaging for surgical-suspicion cases could improve efficiency, reduce costs, and enhance patient outcomes.

Financial implications

From a service perspective, our MRI yield (≈43% normal/degenerative among scans) and low operative conversion (11%) together suggest that routine MRI is often non-actionable in this population. At observed unit costs (£220 new consultation; £163 MRI), the annualised spend for consultations plus MRI alone approximates £288k in our observed flow and ~£451k when scaled to 1,300 annual referrals matching your prior service estimate. If MRI utilisation were reduced from 77.7% to ≈45-50% through selective criteria and earlier physiotherapy assessment, the modelled decrement in scans could yield five- to low six-figure savings annually, even before considering the broader benefits of shorter waits and reduced reattendance [9,10,11]. Additionally, fast-track reporting and review (e.g., structured 7-14-day targets for scanning and sign-off) would directly contract the second diagnostic bottleneck we observed and may be achievable via reporting prioritisation for triaged cases.

**Figure 3:**
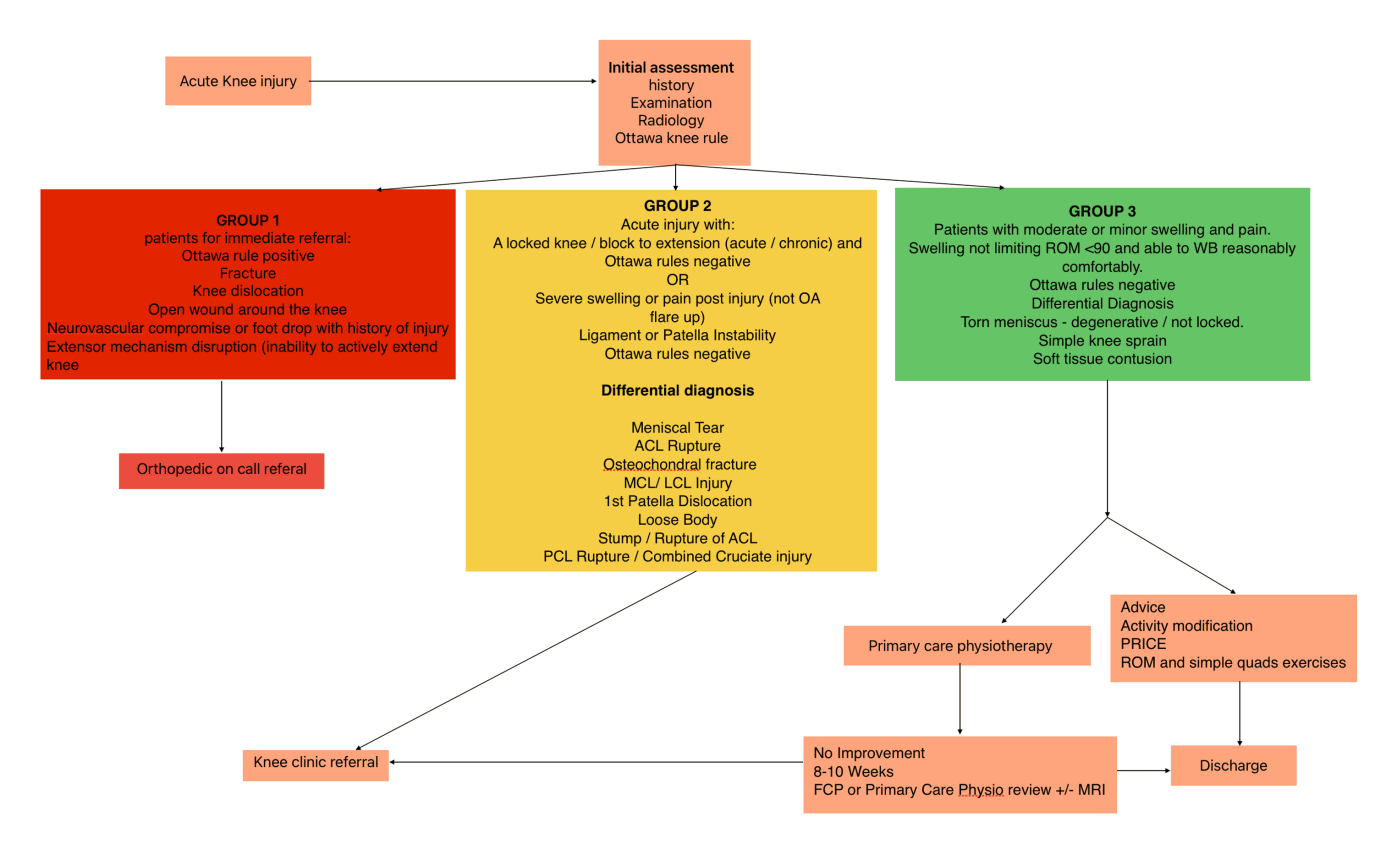
Triage and referral pathway for patients presenting with acute knee injuries This flowchart outlines the structured assessment of acute knee injuries, beginning with initial history, examination, radiology, and application of the Ottawa knee rule. Patients are stratified into three groups: Group 1 (requiring immediate referral, e.g., fracture, dislocation, neurovascular compromise), Group 2 (acute injuries with instability, swelling, or suspected ligament/meniscal pathology), and Group 3 (minor injuries such as soft tissue contusion or simple sprain). Corresponding referral routes include orthopaedic on-call review, knee clinic referral, or primary care physiotherapy with advice and monitoring. Figure created by the authors for this study.

The proposed triage process for patients presenting with acute knee injuries, including referral pathways and differential diagnoses, is summarised in Figure 3.

Strengths and limitations

Strengths include whole-pathway mapping with granular interval analysis, quantitative MRI yield, and pragmatic cost estimation in a real-world NHS setting. Limitations include the retrospective design, missing or implausible dates (necessitating exclusion of negative scans to review intervals from turnaround analysis), and absence of patient-reported outcome measures (PROMs) or indirect cost data (e.g., productivity loss). The findings warrant a prospective re-audit following implementation of the redesigned pathway, incorporating PROMs and economic evaluation.

## Conclusions

The pathway for soft-tissue knee injuries at our trust shows significant delays and suboptimal resource use, especially surrounding MRI and rehabilitation timing. A physiotherapy-first, criteria-based imaging pathway with streamlined reporting/review is likely to shorten time to diagnosis, improve outcomes, and reduce costs consistent with national direction.

## References

[1] Ferry T, Bergström U, Hedström EM (2014). Epidemiology of acute knee injuries seen at the emergency department at Umeå university hospital, Sweden, during 15 years. Knee Surg Sports Traumatol Arthrosc.

[2] Maniar N, Verhagen E, Bryant AL, Opar DA (2022). Trends in Australian knee injury rates: An epidemiological analysis of 228,344 knee injuries over 20 years. Lancet Reg Health West Pac.

[3] Mancino F, Gabr A, Plastow R, Haddad FS (2023). Anterior cruciate ligament injuries in female athletes. Bone Joint J.

[4] Hamer M, Lavoie KL, Bacon SL (2014). Taking up physical activity in later life and healthy ageing: the English longitudinal study of ageing. Br J Sports Med.

[5] Langhammer B, Bergland A, Rydwik E (2018). The importance of physical activity exercise among older people. Biomed Res Int.

[6] Maher NJ, Brogden C, Redmond AC (2025). Disparity in anterior cruciate ligament injury management: a case series review across six National Health Service trusts. BMC Musculoskelet Disord.

[7] Allott NE, Banger MS, McGregor AH (2022). Evaluating the diagnostic pathway for acute ACL injuries in trauma centres: a systematic review. BMC Musculoskelet Disord.

[8] Mohamed A, Babikir E, Mustafa MK (2020). Ottawa knee rule: investigating use and application in a tertiary teaching hospital. Cureus.

[9] Titheradge R, Fisher S, Spalvieri S Virtual fracture clinics: Improving the time to diagnosis following soft tissue knee injuries. Physiotherapy.

[10] Jibuike OO, Paul-Taylor G, Maulvi S (2003). Management of soft tissue knee injuries in an accident and emergency department: the effect of the introduction of a physiotherapy practitioner. Emerg Med J.

[11] Martin L, Liptrot M, Mobberley H CSP2023: 200 - ‘It will get better soon’ A retrospective cohort study of the digital physiotherapeutic management of Emergency Department soft tissue injuries. Physio.

[12] Ahmadi O, Motififard M, Heydari F (2024). The predictive value of point-of-care ultrasonography versus magnetic resonance imaging in assessing medial meniscal tears in patients with acute knee injury. Clin Exp Emerg Med.

[13] (2025). NHS England: Guide to NHS waiting times in England. https://www.nhs.uk/nhs-services/hospitals/guide-to-nhs-waiting-times-in-england.

[14] (2025). NHS England: Diagnostics waiting times and activity (DM01). January.

[15] Ahmed I, Radhakrishnan A, Khatri C (2021). Meniscal tears are more common than previously identified, however, less than a quarter of people with a tear undergo arthroscopy. Knee Surg Sports Traumatol Arthrosc.

[16] D Kim, G Neal-Smith, Wood A (2021). 880 Improving the acute knee injury pathway - a regional quality improvement project in the Oxford university hospitals. Bri Jr Sur.

[17] (2025). NICE: Knee pain assessment. https://cks.nice.org.uk/topics/knee-pain-assessment/.

[18] Academy of Medical Royal Colleges (2025). Academy of Medical Royal Colleges: Knee MRI when symptoms are suggestive of osteoarthritis. AOMRC.

[19] (2025). NHS England: Musculoskeletal orthopaedic approach to referral optimisation. https://www.england.nhs.uk/long-read/msk-orthopaedic-approach-to-referral-optimisation/.

[20] (2025). British Orthopaedic Association: BOASt - best practice for management of anterior cruciate ligament (ACL) injuries. BOA.

